# Genomic characterization and recombination analysis of hepatitis E virus in humans and swine across Asia: implications for food safety

**DOI:** 10.3389/fmicb.2026.1744587

**Published:** 2026-03-11

**Authors:** Dingyu Liu, Zhenwen He, Qin Luo, Baoling Liu, Pian Zhang, Jing Chen, Xiaohu Wang, Gang Wang, Yuan Huang, Hua Xiang, Rujian Cai

**Affiliations:** 1 Guangdong Province Key Laboratory of Livestock Disease Prevention, Scientific Observation and Key Laboratory for Prevention and Control of Avian Influenza and Other Major Poultry Diseases, Ministry of Agriculture and Rural Affairs, Institute of Animal Health, Guangdong Academy of Agricultural Sciences, Guangzhou, China; 2 College of Veterinary Medicine, Huazhong Agricultural University, Wuhan, China; 3 Dongguan Zhongtang Town Agricultural Technical Service Center, Dongguan, China

**Keywords:** Asia, genetic recombination, genetic variation, hepatitis E virus, phylogenetic analysis, zoonoses

## Abstract

**Introduction:**

Hepatitis E virus (HEV) is a globally prevalent zoonotic pathogen posing major public health risks. Swine, a major meat source, carry HEV strains genetically similar to those in humans, highlighting the risk of zoonotic foodborne transmission. This study aimed to investigate the evolutionary history of HEV through phylogenetic and recombination analyses, further provide key reference bases for public health management, improve food safety standards, and offer support for developing effective strategies to prevent foodborne hepatitis E infections.

**Methods:**

We analyzed 348 full-length genomes of HEV isolated from humans and pigs in Asia over the past three decades. Phylogenetic analysis was conducted using the neighbor-joining method in MEGA11. Recombination analysis was performed with seven methods in RDP4, and sequence similarity was visualized using Simplot.

**Results:**

HEV-4 predominated in Asia, especially China, whereas HEV-3 was regionally endemic. Through genomic analysis, we identified 34 potential natural recombination events, predominantly occurring in the RNA-dependent RNA polymerase (RdRp) region; 14 events occurred between swine and human strains, supporting the hypothesis of cross-species transmission. Moreover, 20 recombination events occurred in China and mainly involved HEV-4 strains, suggesting that HEV has distinct evolutionary dynamics. The detection of five inter-genotypic recombination events may highlight ongoing genetic exchange within HEV populations in Asia, and the biological significance of these events remains to be determined.

**Discussion:**

These findings highlight the importance of tracking the evolutionary dynamics of HEV through genomic surveillance, and further underscore the necessity of conducting ongoing HEV surveillance and research to inform prevention strategies.

## 1 Introduction

Human–animal coexistence and anthropogenic activities are major drivers of the emergence and spread of zoonotic diseases worldwide. Zoonosis is characterized by the ability of pathogens to cross species barriers, transmitting from animals to humans. According to the World Health Organization (WHO), approximately 60% of emerging infectious diseases in humans are zoonotic in origin (Rahman et al., 2020). Approximately 25% of these diseases originate from domestic animals, and viruses are responsible for approximately 30% of all zoonotic infections (Tomori and Oluwayelu, 2023).

Hepatitis E, a zoonotic disease caused by hepatitis E virus (HEV), is the most common cause of acute enterically transmitted hepatitis globally (Kamar et al., 2012). HEV is primarily transmitted through the fecal-oral route or through direct contact with infected animals (Meng et al., 2002). The virus was first identified in 1983 by Balayan et al., who characterized it as a non-A, non-B hepatitis virus through electron microscopy of stool samples (Balayan et al., 1983). Since its discovery, HEV has been recognized as endemic in numerous countries, highlighting its global public health relevance.

HEVs belong to the family Hepeviridae, which is divided into the subfamilies Orthohepevirinae and Parahepevirinae. Orthohepevirinae includes four genera: *Paslahepevirus*, which infects humans and mammals; *Rocahepevirus*, which infects rodents; *Chirohepevirus*, which infects bats; and *Avihepevirus*, which infects birds. Parahepevirinae includes just one genus, *Piscihepevirus*, which infects fish (Purdy et al., 2022). *Paslahepevirus* is subdivided into two species: *P. balayani* and *P. alci*. HEVs are included under the species *P. balayani* and are regarded as having a single serotype with eight genotypes: HEV-1-HEV-8, with 36 subtypes (Smith et al., 2020). The distribution of these different HEV genotypes varies globally, and they are associated with different hosts. HEV-1 and HEV-2 are found only in humans and are transmitted through water sources. HEV-1 is endemic to West Africa, North Africa, and Asia, whereas HEV-2 is endemic to Asia, Africa, and Mexico (Raji et al., 2022). HEV-3, the most common HEV genotype globally, is widely distributed in developed countries and some developing countries. HEV-3 has been found in humans and various animals, and is primarily transmitted through food (Sridhar et al., 2017). HEV-4 is mainly found in Asia, especially in China, Japan, Vietnam, and Thailand. It is also zoonotic and foodborne (Aggarwal, 2011). HEV-5 and HEV-6 have been identified in wild boars in Africa (Khuroo, 2023). HEV-7 and HEV-8 are mainly found in camels in the Middle East (El-Kafrawy et al., 2020).

The increasing number of hepatitis E cases globally underscores the zoonotic potential of HEV. Initially considered endemic only to developing countries, HEV infections are now being reported from developed countries such as the UK, USA, and Japan. In these regions, the primary transmission route has shifted from travel-associated exposure to the consumption of HEV-contaminated animal products, particularly porcine-derived foods. Pigs, which are recognized as the main reservoirs of HEV, typically exhibit subclinical infections. Following a 1–2-week incubation period, infected pigs enter a viremic phase characterized by fecal viral shedding lasting 7–50 days. After viraemia, pigs cease to transmit the virus to their conspecifics; however, HEV RNA persists in the porcine liver, bile, and other organs, even after clinical resolution (Krasochko et al., 2022; Carella et al., 2023). Consumption of HEV RNA-positive pork products by humans constitutes a major pathway for zoonotic transmission. In Asia, the seroprevalence of swine HEV (sHEV) antibodies ranges from 35.0 to 73.0%, with RNA detection rates of 5.0–46.0% (He et al., 2024). Recent studies provide robust evidence of the genetic relatedness between sHEV and human HEV strains, confirming the pivotal role of pigs in HEV transmission to humans (Nishizawa et al., 2003; Choi et al., 2003; Conlan et al., 2011).

Genetic recombination among HEV strains may play a critical role in facilitating cross-species transmission. Recombination can occur when strains from different hosts infect the same individual, potentially producing progeny viruses with novel host specificities and enhancing bidirectional zoonotic transmission (animal-to-human or human-to-animal). Several natural recombination events have been documented in HEV evolution. In 2005, van Cuyck et al. (2005) provided the first evidence of recombination among different HEV strains; however, the phylogeny and recombination of only 32 complete genomes were analyzed. In 2009, Fan (2009) revealed inter-genotypic recombinants through open reading frame (ORF) structure analysis. In 2012, Chen et al. (2012) found that recombination events occurred non-randomly, with the highest frequencies observed in the X structural domain and deconjugation enzymes. Furthermore, in 2021, Shen et al. (2021) analyzed 557 full-length HEV genomes and identified four major recombination events—three in ORF2 and one in ORF1—highlighting recurrent hotspots. However, limited attention has been given to recombination between human and porcine strains in Asia. According to statistics from the Organization for Economic Co-operation and Development, global pork production and consumption reached 124,864 tons in 2024, with Asia accounting for 55.1% of production (68,848 tons) and 59.0% of consumption (73,688 tons). China alone is responsible for 46.5% of production (58,063 tons) and 47.8% of consumption (59,648 tons) (OECD, 2024). As the leading producer and consumer of pork, Asia plays a central role in shaping the epidemiology of HEV. The dense human–swine interface, fueled by extensive farming and pork consumption in Asia, may contribute to high genetic homology between swine and human HEV strains. This close interspecies contact may also facilitate genetic recombination events between porcine and human HEV variants, and potentially increase the risk of zoonotic transmission.

Given the central role of pigs in Asian food system and their contact with humans, the widespread circulation of HEV in swine poses major public health and food safety challenges. To address these challenges, it is essential to assess the distribution and genetic characteristics of emerging HEV strains. In this study, we focused on screening and analyzing the whole-genome sequences of HEV strains in Asia, while aiming to elucidate their molecular characteristics. The purpose of this experiment is to investigate the evolutionary history of HEVs by conducting phylogenetic and recombination analyses, provide key reference bases for public health management, improve food safety standards, and meanwhile offer support for the development of effective strategies to prevent foodborne hepatitis E infections.

## 2 Materials and methods

### 2.1 Dataset

Sequences were retrieved from the NCBI Virus database^1^ using the search term “*Paslahepevirus balayani*, taxid:1678143.” Filters were applied to include only sequences with a release date between 1990 and 1 April 2024, nucleotide completeness set to “complete,” and geographic region specified as “Asia.” Since our study focuses on human populations and swine populations, we therefore excluded sequences from non-human hosts and non-swine hosts—swine hosts include domestic swine and wild boar. Sequence alignment was performed using DNAstar, followed by a redundancy filter to remove entries sharing >99.9% nucleotide similarity across the entire genome. This 99.9% threshold was selected to minimize oversampling of near-identical strains from the same outbreak, farm, or epidemiologically linked source, which could artificially inflate recombination event counts. A summary of the number of sequences removed per genotype and country is provided in Supplementary Figure 1. The resulting sequences were used in the subsequent analyses.

### 2.2 Phylogenetic analysis

Phylogenetic relationships were inferred using the neighbor-joining method (Saitou and Nei, 1987) based on the amino acid sequences of the full genome. The percentage of replicate trees in which the associated taxa clustered together in the bootstrap test (1,000 replicates) is shown next to the branches (Felsenstein, 1985). The tree was drawn to scale, with branch lengths in the same units as those used to calculate evolutionary distances. The evolutionary distances were computed using the p-distance method; they are expressed as the number of amino acid differences per site. The p-distance model was chosen for its direct quantification of differing site proportions, high computational efficiency, and straightforward interpretation—ideal for our study’s HEV strains with high sequence homology. All phylogenetic analyses were conducted using MEGA11 software (Tamura et al., 2021).

### 2.3 Recombination analysis

Full-length HEV genomes were subjected to recombination analysis using seven different methods implemented in RDP4 (version 4.101): RDP, GENECONV, Bootscan, 3Seq, Chimaera, SiScan, and MaxChi. Recombination events with *p*-value <0.05 in any of the methods were considered significant (Martin et al., 2017). To assess whether the observed difference in the number of recombination events involving different genotype sequences was statistically significant, a comparative analysis of recombination participation rates was performed. Each complete genome sequence was assigned a binary status: it was considered “involved in recombination” if it was identified as either a recombinant or a parental strain in any recombination event detected by RDP4; otherwise, it was categorized as “not involved.” The two-tailed Fisher’s exact test was applied to compare the proportions of sequences involved in recombination between the two genotypes. This test was implemented using GraphPad Prism software (version 9.5). A *p*-value of <0.05 was considered statistically significant. To visualize sequence similarity, Simplot software (version 3.5.1) was used with the following parameters: 200 bp; step size, 20 bp; T/t ratio, 2.0; and nucleotide substitution model, Kimura (2-parameter) (Martin et al., 2005).

## 3 Results

### 3.1 Sequence search results

A total of 410 full-length genome sequences of HEV from Asia were retrieved from the NCBI GenBank database via the NCBI Virus website. Sequences associated with non-human and non-porcine hosts were excluded from the initial dataset. Subsequently, DNASTAR was used to perform sequence matching, and sequences exhibiting greater than 99.9% similarity were removed to ensure genetic diversity. The final dataset, comprising 348 isolates from 8 countries, was used for downstream analyses (Supplementary Figure 1; Supplementary Table 1).

### 3.2 Phylogenetic analysis of 348 complete HEV genome sequences from Asia

**Figure 1:**
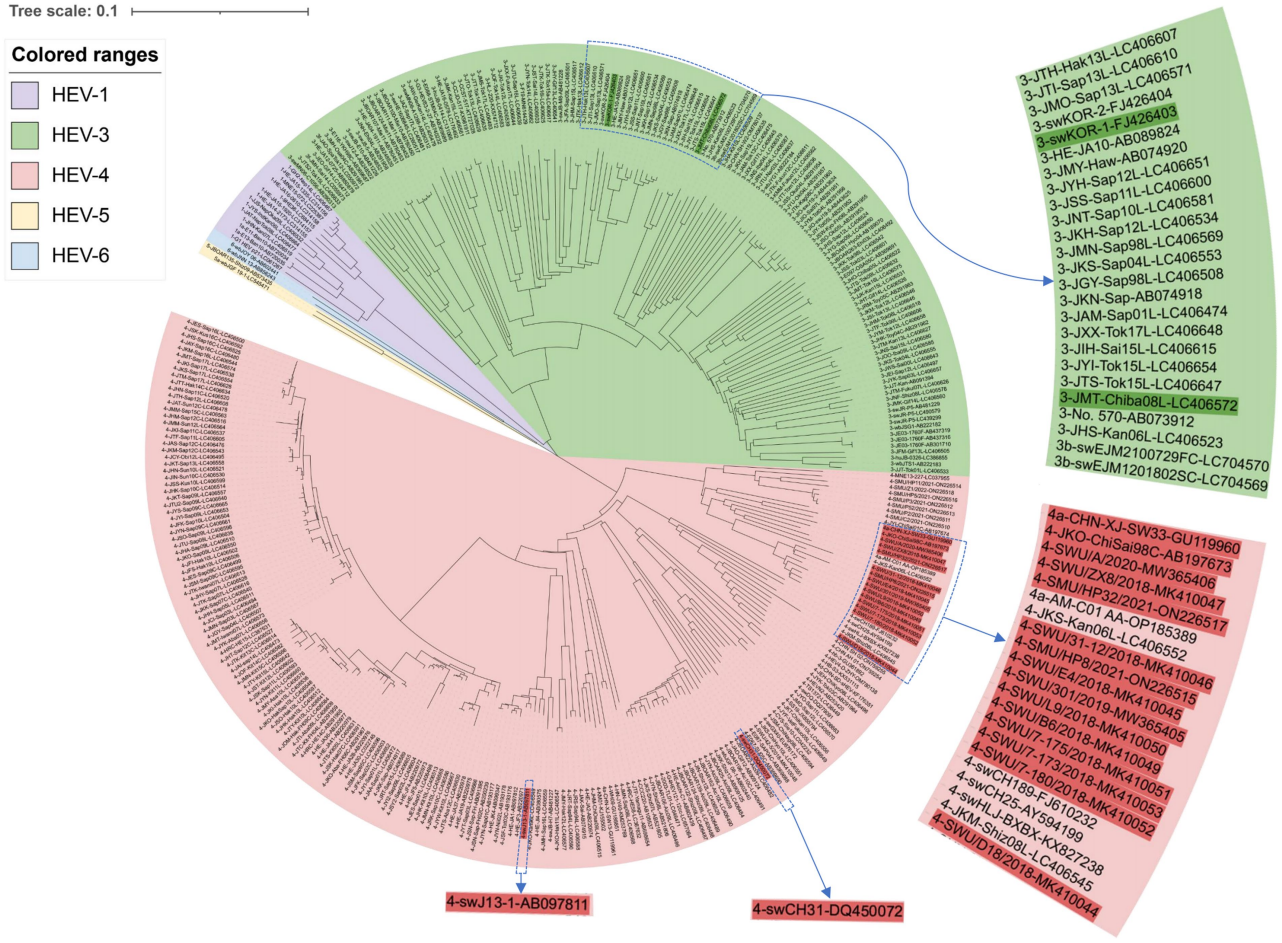
Phylogenetic analysis of 348 HEV strains in Asia.

The phylogenetic tree analysis of the 348 HEV sequences revealed the presence of *Orthohepevirus* A subtypes, HEV-1 (14/348), HEV-3 (134/348), HEV-4 (196/348), HEV-5 (2/348), and HEV-6 (2/348) sequences (Figure 1).

HEV-1, which infects only humans, was represented by 14 sequences from five countries: Japan (9/14), Mongolia (1/14), India (1/14), Nepal (1/14), and Bangladesh (2/14). HEV-3 and HEV-4, which are zoonotic genotypes, infect humans and pigs. Among the 134 HEV-3 sequences, 2 were from Mongolia, 125 from Japan, 3 from South Korea, and 4 from China. The 196 HEV-4 sequences were from Mongolia (1/196), Japan (150/196), China (44/196), and Cambodia (1/196). Two HEV-5 and two HEV-6 sequences were detected in wild boar samples from Japan (Supplementary Figure 2).

### 3.3 Recombination analysis

**Figure 2:**
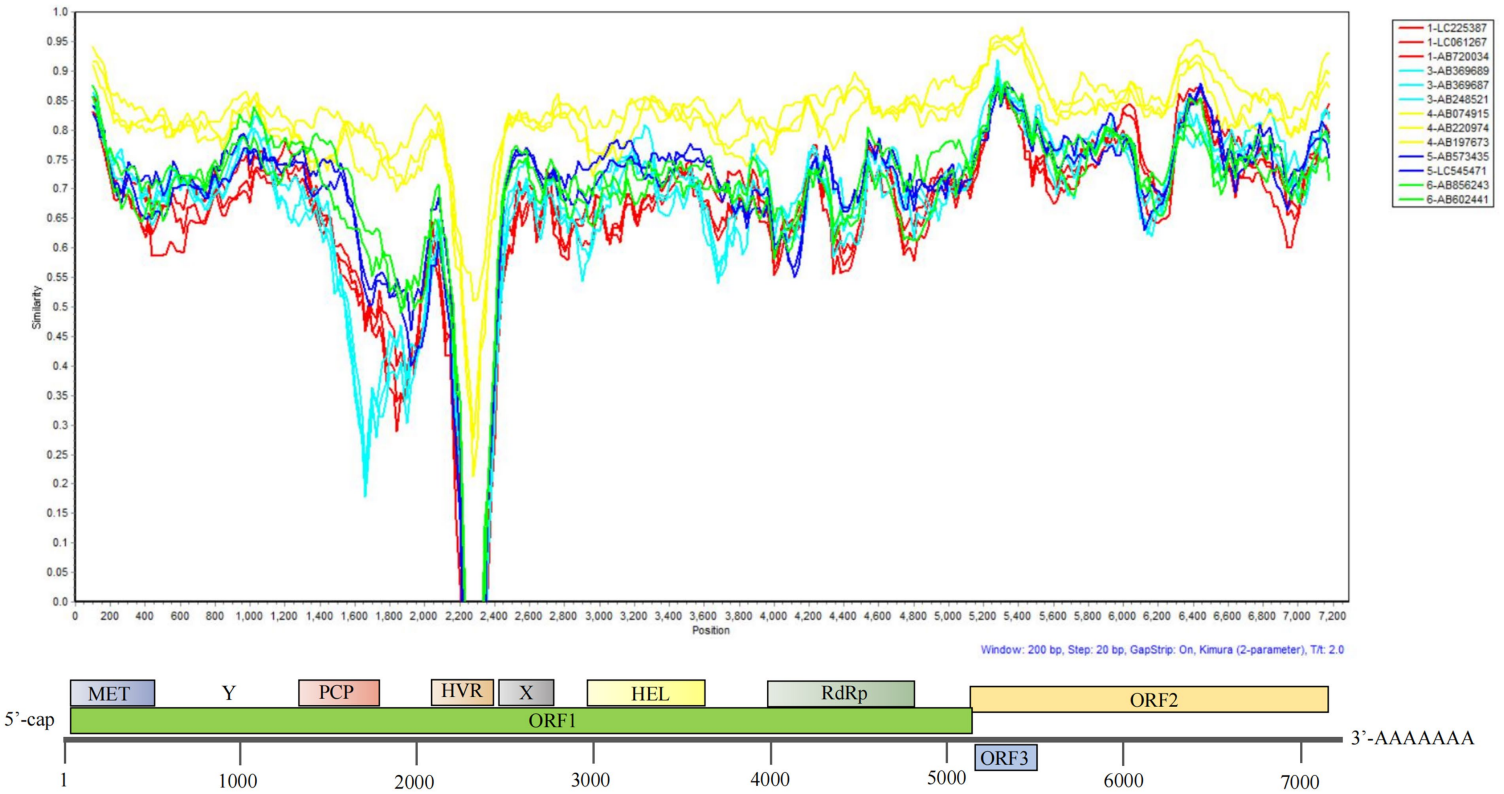
Genomic similarity analysis of 13 representative HEV isolates across genotypes in Asia.

To further investigate the genetic characteristics of HEV strains in Asia, we performed genomic similarity analysis using 13 representative full-length genomes: three strains each of HEV-1, HEV-3, and HEV-4, and two strains each of HEV-5 and HEV-6 (Figure 2). The complete swDQ genome sequence (GenBank ID: DQ279091, host: swine; genotype: HEV-4) was used as the query. The selection of strain DQ279091 as the query sequence is based on its well-recognized status as a representative sequence of HEV-4 (Smith et al., 2020), which is highly aligned with the core objective of this study to focus on the evolution and recombination of HEV in the Asian region, given that HEV-4 is the predominant genotype among the Asian HEV sequences identified in our study (196/348). The lowest genetic similarity was observed in the hypervariable region of the ORF1 coding region, whereas the ORF3/ORF2 region exhibited the highest sequence conservation. Consistent with the phylogenetic analysis results, the HEV-1, HEV-3, HEV-4, HEV-5, and HEV-6 strains clustered into distinct genomic similarity groups.

**Figure 3:**
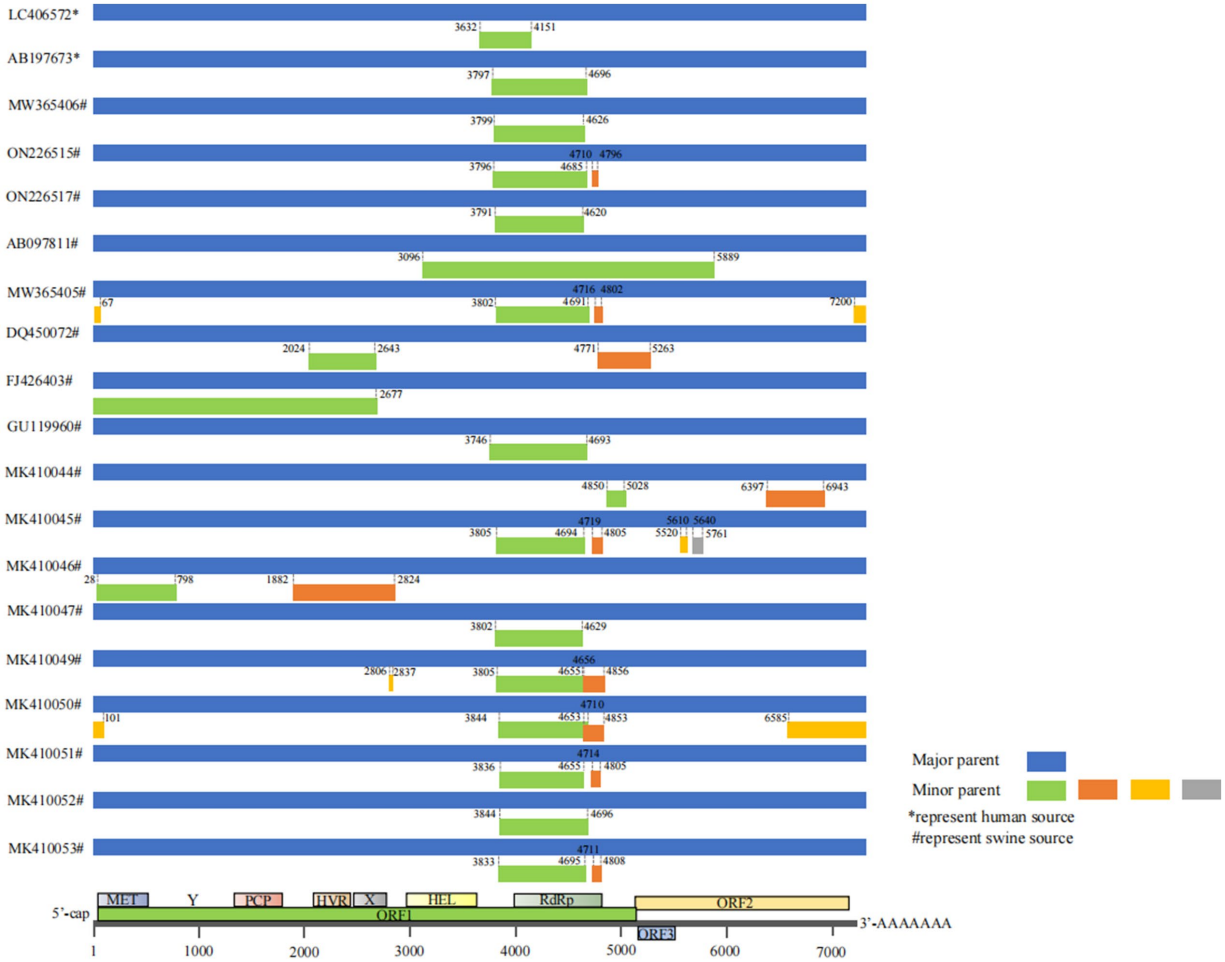
Genomic structure of HEV and recombination map of HEV genomes.

We analyzed 348 full-length HEV genome sequences and identified 34 potential recombination events (Figure 3). Among these, 29 were intra-genotypic and 5 were inter-genotypic recombination events. Geographically, 22 events occurred between isolates from the same country, whereas 12 events involved isolates from different countries. Among the 34 recombination events, 18 (52.94%) occurred between swine-derived HEV strains, 2 (5.88%) occurred between human-derived strains, and 14 (41.18%) involved cross-species recombination between swine and human (Table 1). Furthermore, 27 recombination events (79.41%) occurred within HEV-4. Among the 196 HEV-4 sequences, 28 sequences (14.29%) were involved in recombination events, whereas 10 out of 134 HEV-3 sequences (7.46%) participated in recombination. The observed difference in the number of recombination events between HEV-4 and HEV-3 was formally tested by comparing the proportions of sequences involved in recombination for each genotype. The two-tailed Fisher’s exact test indicated that this difference in proportion was not statistically significant (*p* = 0.078) (Supplementary Table 1).

**Table 1:** Detailed information on potential recombination events identified in the full-length genomes of hepatitis E virus (HEV) isolated between 1993 and 2021.

Event No.	Recombinant	Major parent	Minor parent
	GenBank ID (host-country-year-genotype)	GenBankID (host-country-year-genotype)	GenBankID (host-country-year-genotype)
1	AB197673 (Human-Japan-1998-4)	MK410049 (Swine-China-2018-4)	MK410046 (Swine-China-2018-4)
2	AB097811 (Swine-Japan-2001-4)	AB193177 (Human-Japan-2001-4)	AB481227 (Swine-Japan-2008-4)
3	DQ450072 (Swine-China-2005-4)	AB369690 (Human-Japan-2005-4)	GU188851 (Swine-China-2009-4)
4		AB369690 (Human-Japan-2005-4)	OM780137 (Human-China-2017-3)
5	FJ426403 (Swine-SouthKorea-2007-3)	LC406508 (Human-Japan-1998-3)	FJ426404 (Swine-South Korea-2007-3)
6	LC406572 (Human-Japan-2008-3)	LC406523 (Human-Japan-2006-3)	LC406546 (Human-Japan-2012-3)
7	GU119960 (Swine-China-2009-4)	MK410049 (Swine-China-2018-4)	MK410046 (Swine-China-2018-4)
8	MK410044 (Swine-China-2018-4)	AY594199 (Swine-China-2003-4)	LC406664 (Human-Japan-2006-1)
9		KX531115 (Swine-China-2014-4)	AB197673 (Human-China-1998-4)
10	MK410045 (Swine-China-2018-4)	MK410049 (Swine-China-2018-4)	MK410046 (Swine-China-2018-4)
11		ON226518 (Swine-China-2022-4)	MK410052 (Swine-China-2018-4)
12		MW365405 (Swine-China-2019-4)	LC406533 (Human-Japan-2001-3)
13		ON226515 (Swine-China-2021-4)	AB197673 (Human-China-1998-4)
14	MK410046 (Swine-China-2018-4)	ON226516 (Swine-China-2021-4)	MK410045 (Swine-China-2018-4)
15		ON226513 (Swine-China-2021-4)	MK410045 (Swine-China-2018-4)
16	MK410047 (Swine-China-2018-4)	MK410049 (Swine-China-2018-4)	MK410046 (Swine-China-2018-4)
17	MK410049 (Swine-China-2018-4)	MK410050 (Swine-China-2018-4)	LC406497 (Human-Japan-2012-3)
18		LC406552 (Human-Japan-2006-4)	MK410046 (Swine-China-2018-4)
19		MK410052 (Swine-China-2018-4)	ON226518 (Swine-China-2022-4)
20	MK410050 (Swine-China-2018-4)	MW365405 (Swine-China-2019-4)	EU676172 (Swine-China-2007-4)
21		LC406552 (Human-Japan-2006-4)	MK410046 (Swine-China-2018-4)
22		MK410052 (Swine-China-2018-4)	ON226518 (Swine-China-2022-4)
23	MK410051 (Swine-China-2018-4)	LC406552 (Human-Japan-2006-4)	MK410046 (Swine-China-2018-4)
24		ON226518 (Swine-China-2022-4)	MK410052 (Swine-China-2018-4)
25	MK410052 (Swine-China-2018-4)	LC406552 (Human-Japan-2006-4)	MK410046 (Swine-China-2018-4)
26	MK410053 (Swine-China-2018-4)	LC406552 (Human-Japan-2006-4)	MK410046 (Swine-China-2018-4)
27		ON226518 (Swine-China-2022-4)	MK410052 (Swine-China-2018-4)
28	MW365405 (Swine-China-2019-4)	MK410049 (Swine-China-2018-4)	LC164712 (Human -Japan-2014-3)
29		MK410049 (Swine-China-2018-4)	MK410046 (Swine-China-2018-4)
30		ON226518 (Swine-China-2022-4)	MK410052 (Swine-China-2018-4)
31	MW365406 (Swine-China-2020-4)	MK410049 (Swine-China-2018-4)	MK410046 (Swine-China-2018-4)
32	ON226515 (Swine-China-2021-4)	MK410049 (Swine-China-2018-4)	MK410046 (Swine-China-2018-4)
33		ON226518 (Swine-China-2022-4)	MK410052 (Swine-China-2018-4)
34	ON226517 (Swine-China-2021-4)	MK410049 (Swine-China-2018-4)	MK410046 (Swine-China-2018-4)

## 4 Discussion

The results of our comprehensive analysis of full-length HEV genome sequences isolated from humans and pigs in Asia over the past three decades provide valuable insights into the genetic characteristics, evolutionary patterns, and potential risks associated with HEV, particularly in China. HEV-3 is endemic to the Americas, Europe, and parts of Asia; whereas, HEV-4 is predominantly endemic to Asia, including China, Japan, and Korea (Wang et al., 2016; Jeong et al., 2017). We retrieved 134 HEV-3 sequences—125 from Japan and 9 from China, Korea, and Mongolia. Among the 196 HEV-4 sequences retrieved, 150 were from Japan, 44 from China, and 2 from Mongolia and Cambodia. These findings, consistent with a previous report (Aggarwal, 2011), confirm the predominance of HEV-4 strains in Asia, particularly in Japan and China. The dominance of this genotype in China can be attributed to various factors, including its transmission through foodborne routes. The constructed phylogenetic trees further supported this observation and showed a distinct clustering of HEV-4 strains isolated from both humans and pigs. The greater number of HEV-4 sequences from Japan may reflect more extensive epidemiological surveillance and genome amplification. Given the critical role of genomic data in understanding HEV evolution and transmission, the development of a comprehensive database of complete or sub-genomic HEV sequences should be a priority in epidemiological research. HEV-5 and HEV-6 are genotypes found in Japanese wild boar (Takahashi et al., 2011; Sato et al., 2011). These genotypes clustered closely in our phylogenetic analyses (Figure 1), suggesting potential evolutionary links. This finding underscores the importance of monitoring HEV in wild animal populations as part of broader control efforts.

A key finding of our study was the identification of multiple potential natural recombination events in the HEV genomes, with a significant proportion occurring in China. In 2022, Bahoussi et al. analyzed and compared the full-length genomes of 40 sHEV isolates from China over the past two decades and identified 8 potential natural recombination events, 4 of which occurred in China (Bahoussi et al., 2022). In contrast, we analyzed 348 full-length genomes, including porcine strains from China and other Asian countries and human sources. We identified 34 potential recombination events, of which 20 occurred only in China and all were HEV-4. Similar to the findings of Bahoussi et al., recombination occurred mainly between HEV-4 strains. Viral recombination is the exchange of genetic material between two or more different viral strains within the same host cell resulting in novel variants with altered pathogenicity, transmissibility, or host range (Bentley and Evans, 2018). Therefore, recombination is a key process in viral evolution that can lead to viruses acquiring new genetic traits that can improve their fitness, virulence, or transmission efficiency. RNA viruses such as HEVs are particularly susceptible to mutations as a result of genetic recombination. For example, Wang et al. (2024) identified a potential recombination event in the S gene of porcine epidemic diarrhea virus strains that may have accelerated its evolution. Hu et al. (2024) found that recombination of the S2 subunit of porcine delta coronavirus altered the pathogenicity and organogenesis of the strain. We hypothesized that the frequent occurrence of HEV recombination events in China may be attributed to its intensive swine industry characterized by high-density pig farming operations, large-scale pork consumption, and prolonged human–swine interactions. The high recombination frequency of HEV-4 may also be associated with its biological characteristics. A study by Imagawa et al. (2018) found that HEV-4 exhibits slightly higher thermal stability than HEV-3, enabling it to survive longer in water, feed, or contaminated pork products. This prolonged environmental persistence increases the exposure probability between different hosts and viral strains, providing a prerequisite for co-infection and recombination. Regarding the statistical comparison of recombination frequency between HEV-4 and HEV-3, our formal analysis using Fisher’s exact test did not yield a statistically significant difference in the proportion of sequences involved in recombination (*p* > 0.05). This outcome likely reflects, in part, the inherent limitations of the available sequence dataset, including uneven sample sizes across genotypes and the relatively low overall frequency of recombination events, which reduces statistical power to detect differences. Although our study did not conduct a detailed subtype phylogenetic analysis, we still observed that strains of subtype 4a are the dominant recombinant strains, which may also be related to the high prevalence and wide distribution of subtype 4a (Smith et al., 2020). Furthermore, among the potential recombination events identified in this study, a large sampling time span was observed between some major and minor parent strains. This phenomenon reflects that older HEV lineages may not be naturally eliminated and persist in the transmission chain. Older and newer strains may undergo co-infection across different time scales through human mobility and inter-regional transportation of live pigs. Recombination between conserved genetic fragments carried by older strains and newly circulating strains may produce recombinant variants with both environmental adaptability and host adaptability. Although the core driver of cross-temporal recombination is the long-term persistence of older strains, the impact of sampling bias cannot be completely excluded. However, considering human mobility, farming and trade patterns in Asia, we believe that older lineages may undergo long-term circulation. Such recombination events further confirm the complexity of the HEV transmission chain and highlight its strong adaptability as a zoonotic virus. Furthermore, the higher frequency of HEV-4 recombination events in China may also be attributable to heterogeneity in sampling strategies. Chinese samples may be more concentrated in high-risk settings, such as intensive farming zones, provinces with high HEV prevalence, and slaughterhouse traceability samples (He et al., 2024). In these environments, opportunities for viral exposure and cross-infection are heightened, making recombination events more likely to be detected. Geographical isolation may also contribute to variations in recombination frequency. China’s vast territory facilitates more frequent inter-provincial movement of farmed pigs, potentially promoting mixing and recombination of different HEV strains. In contrast, Japan’s insular geography results in relatively closed pig populations with limited genetic exchange between strains, naturally reducing recombination probability.

Recombination may potentially facilitate cross-species transmission. For instance, Chen et al. isolated recombinant strains from pigs in China in 2006 that were genetically linked to human HEV strains from Japan (Chen et al., 2012). Recombination events enhance HEV genetic diversity, thereby potentially generating novel viral strains with enhanced host adaptability that facilitate cross-species transmission. Concurrently, recombinant strains may acquire traits favoring survival and dissemination within new hosts and undergo continuous adaptive evolution under natural selection conditions (Pérez-Losada et al., 2015). In our study, 14 of the 34 identified HEV recombination events (41.18%) involved both human and pig HEV strains. All 14 recombinant strains were isolated from swine hosts. Among them, the major parents of 8 recombinant strains were of human origin, while those of the remaining 6 strains were of swine origin. When the major parent was derived from humans, the most plausible transmission route was foodborne: HEV-infected individuals consumed HEV-contaminated pork or pork products, leading to the occurrence of recombination events within the human body. However, for the transmission of human-derived recombinant strains to swine herds that enables the concurrent presence of human and swine HEV strains in swine hosts, the most plausible underlying pathway is direct contact in the farming environment. Farm workers, particularly those in pig farming environments, are at increased risk of exposure to HEV. The prevalence of HEV among farm workers could be higher in rural areas, where large-scale swine farms are more common, leading to greater HEV presence in these environments, suggesting that water and food used in pig farms may not be adequately treated, potentially becoming contaminated with human-derived HEV strains, which could result in recombination between human and pig strains of the virus (Aggarwal et al., 2000; Geng et al., 2016). Swine are highly susceptible to HEV, which is primarily transmitted via the fecal-oral route; thus, consumption of contaminated drinking water or feed enables swine to ingest human-derived HEV, subsequently establishing infection in their bodies (Kasorndorkbua et al., 2004; Pavio et al., 2017). Notably, 11 of these 14 cross-host events occurred among strains from different countries, indicating that HEV may undergo genetic recombination after spreading across regions to adapt to new environments and potentially gain the capacity to infect new hosts. These findings suggest that infected swine or processed pork products transported across regions could facilitate zoonotic transmission through recombination events. Therefore, increased vigilance is warranted regarding HEV contamination of imported/exported food products, which may serve as vehicles for cross-regional viral dissemination.

The recombination events in HEV were not randomly distributed across the genome; they occurred at a high frequency in specific loci, notably the X structural domains and deconjugase, and RNA-dependent RNA polymerase (RdRp) regions. This observation supports previous study findings that highlight the role of ORF1 in viral adaptations (Chen et al., 2012). Similar patterns were observed in our study: among the 34 potential recombination events, 22 (64.71%) involved the RdRp region. Given that RdRp is essential for viral genome replication, recombination in this region may substantially influence viral adaptability. Chen et al. (2012) identified the X domain, helicase, and RdRp as recombination hotspots during their analysis of 160 complete HEV genome sequences. Similar to our findings, but differing in that three recombination breakpoints were located within the methyltransferase domain (MET) in our study. MET participates in viral RNA 5′-end capping modification, maintaining RNA stability and translation efficiency, and is a key enzyme in viral replication. Based on Chen et al.'s (2012) natural selection analysis of 170 HEV sequences, 97.25% of codons in this region undergo strong negative selection, indicating highly conserved function. Any amino acid mutation may disrupt its core enzymatic activity. Mutations in MET significantly impair HEV’s replication capacity, RNA stability, and host adaptability, likely leading to reduced pathogenicity or natural selection elimination. Therefore, recombination does not necessarily confer enhanced adaptability to HEV; it may instead precipitate natural elimination. The precise outcome of recombination necessitates comprehensive understanding of all functional domains within the HEV genome. Furthermore, analysis of codon usage similarity between the HEV genome and its host genome using the relative codon de-optimization index (RCDI) revealed that HEV-3 and HEV-4 had similar RCDI values in humans and wild boars, suggesting a shared adaptation of zoonotic HEVs to these hosts. When comparing the codon adaptation index (CAI) values of different HEV ORFs, ORF1 and ORF3 emerged as likely contributors to host tropism (Wang et al., 2022). In summary, recombination of HEVs may have an important effect on their ability to spread across species, potentially making it easier for viruses to spread among different hosts by increasing their genetic diversity and facilitating new transmission routes.

Cross-genotype transmission of the HEV is well documented (Takahashi et al., 2002; Meng, 2016; Bahoussi et al., 2022). In the present study, we identified five inter-genotype recombination events in Asia. Specifically, four of these events occurred between HEV-3 and HEV-4, with only one event involving HEV-1 and HEV-4. The recombinant strain MK410044 (swine, China, 2018, genotype 4) had AY594199 (swine, China, 2003, genotype 4) as its major parent strain and LC406664 (human Japan, 2006, genotype 1) as its minor parent strain. The recombination breakpoints of this event were identified at positions 6,397 and 6,943, which are located within the ORF2 region of the HEV genome. The ORF2 region encodes the capsid protein of HEV, a core structural component responsible for host cell receptor binding, viral entry into host cells, and induction of neutralizing antibodies in the host (Sayed et al., 2022). Notably, HEV-1 is recognized as a human-restricted genotype; the insertion of its genetic fragment into the ORF2 region of the swine-derived HEV-4 strain may alter the spatial conformation of the capsid protein, thereby theoretically affecting the viral host tropism and adaptability. This alteration may potentially enhance the recombinant strain’s dual recognition capability for both swine and human cell receptors, or improve its immune evasion efficiency during cross-species transmission. The recombinant strain DQ450072, identified as originating from China with pigs as its host, exhibited recombination between HEV-3 and HEV-4 genotypes. This finding is consistent with the cross-genotype recombination observed in studies by Fan (2009) and Chen et al. (2012). However, the nucleotide breakpoint locations differ. Fan (2009) identified breakpoints at 4749–4758 nt and 5,226–5,270 nt, corresponding to ORF1 (RdRp region) and the ORF2-ORF3 overlap region; Chen et al. (2012) identified breakpoints at 1643–2017 nt and 4,132–4,557 nt, corresponding to the ORF1 region. Our findings align with those of Fan (2009), and such discrepancies may stem from variations in detection methods and sequence alignment benchmarks. However, the inter-genotypic recombination identified in our study only involved HEV-1/HEV-4 and HEV-3/HEV-4, and did not involve recombination between HEV-1 and HEV-3 genotypes. This differs from the recombinant strain HEV_32_Manchester_301214 (United Kingdom, HEV-3) identified by Shen et al. (2021). This recombinant strain resulted from inter-genotypic recombination between HEV-3 (major parent HEPAC-44, France) and HEV-1 (minor parent HE-JA15-1335, Japan), both of which were human isolates. This discrepancy may stem from our study’s focus on Asia, which excluded Europe where HEV-3 is more prevalent. Notably, four of these five events involved cross-species recombination between humans and pigs. Therefore, we hypothesized that close contact between humans and pigs not only facilitates intra-genotype recombination but also increases the likelihood of inter-genotype or transgenic recombination. This finding is particularly concerning, as it suggests that HEV can cross genotype boundaries leading to the emergence of new HEV lineages with distinct genetic and epidemiological characteristics. The emergence of new genotypes in previously unrecognized hosts supports this hypothesis. For example, HEV-7 and HEV-8 have been detected in camels (Woo et al., 2014; Woo et al., 2016). Additionally, 27 recombination events occurred among HEV-4 strains, accounting for 79.41% of all identified events, indicating that HEV-4 may be more prone to recombination than other genotypes. Imagawa et al. (2018) compared the thermal stability of HEV-3 and HEV-4 and found that HEV-4 is more thermally stable than HEV-3. This finding suggests that differences in the stability of viral particles may influence environmental persistence, with HEV-4 potentially surviving longer and thus having more opportunities for recombination. These findings highlight the importance of monitoring HEV-4 in particular, given its potential and apparent predisposition to genetic recombination.

In the present study, the genomic similarity analysis using SimPlot further supported the close genetic relationships among HEV strains isolated from different hosts and geographical locations within Asia. The high degree of similarity observed among HEV strains from Asian countries suggests the possibility of sustained cross-species transmission and establishment of a zoonotic cycle. This is particularly concerning in regions where frequent human–animal contact increases the risk of spillover events and potentially lead to the emergence of novel HEV variants with implications for public health. These variants may evade existing diagnostic tools, treatments, and vaccines, thereby posing challenges for effective disease management. Although we did not explore HEV subtypes in detail owing to the complexity of subtype classification, Smith et al. (2016, 2020) analyzed the nucleotide p-distances of all available complete HEV genome sequences and assigned reference sequences to each subtype. They identified seven subtypes of HEV-1 (1a, 1b, 1c, 1d, 1e, 1f, 1 g), including the newly recognized subtype 1 g, which form well-defined phylogenetic clades rather than two evolutionary branches as previously suggested. Excluding the rabbit-associated HEV evolutionary branch (subtype 3ra), they identified 13 subtypes of HEV-3 (3a, 3b, 3c, 3e, 3f, 3 g, 3 h, 3i, 3j, 3 k, 3 L, 3 m) and 9 subtypes of HEV-4 (4a–4i), with the latter exhibiting three distinct subclades within subtypes 4b and 4c without further subdivision. However, a definitive threshold for subtype classification remains elusive because p-distance values form a continuous spectrum within and between subtypes—for example, intra-subtype p-distances of HEV-1 can reach up to 0.06, overlapping with inter-subtype distances (>0.037). Moreover, subtypes have not been consistently associated with increased clinical severity or enhanced cross-species transmissibility (Smith et al., 2015). Our study has several limitations. Firstly, although our dataset comprises 348 HEV sequences from 8 Asian countries, there is a significant bias in the geographical distribution of samples within Asia. Most sequences originated from Japan and China, while samples from other Asian countries were extremely limited, resulting in insufficient representativeness of the study findings for these regions. Therefore, our finding that HEV-4 predominates in Asia and exhibits greater susceptibility to recombination may stem from its higher representation within the dataset (196/348). The underrepresentation of other genotypes of sequences may introduce bias in the detection of recombination events. For instance, only 14 sequences were available for HEV-1, while HEV-5 and HEV-6 each had merely two sequences. Secondly, the host range was limited. Our study focused solely on two host categories—humans and pigs—and did not include other known hosts of HEV, which may lead to an incomplete understanding of the HEV transmission network. Moreover, the recombination events identified in our study lacked validation. Although 34 potential recombination events were detected via bioinformatics methods, no experimental validation of their biological functions was conducted. The absence of supporting evidence means the actual public health risks of these recombination events cannot be accurately assessed.

## 5 Conclusion

This study provides valuable insights into the genetic characteristics, evolutionary patterns, and potential public health risks associated with foodborne transmission of HEV in Asia. Our findings highlight the predominance of HEV-4 across the region, high degree of genomic similarity among HEV strains from different hosts and geographical locations, and frequent occurrence of natural recombination events, particularly in China. These observations underscore the urgent need for continuous HEV monitoring and surveillance. This includes establishing a robust system for collecting, analyzing, and sharing HEV genome sequences and related epidemiological data across countries and regions. Furthermore, strengthening the surveillance and monitoring of HEV in animal populations, particularly pigs, is imperative given that swine are a major source of zoonotic HEV. The high genetic homology observed between porcine and human HEV strains, coupled with the substantial prevalence of cross-species recombination events, underscores the critical role of swine in driving HEV transmission. To mitigate the risk of cross-regional and cross-species HEV dissemination, region-specific surveillance programs should be implemented for HEV in swine populations accompanied by stricter quarantine measures for live pigs and pork products in international trade. These findings have important implications for the public health governance of hepatitis E and the development of preventive strategies to ensure meat safety.

## Data Availability

Publicly available datasets were analyzed in this study. This data can be found at: https://www.ncbi.nlm.nih.gov/labs/virus/vssi/#/.

## Footnotes

1
https://www.ncbi.nlm.nih.gov/labs/virus/vssi/#/
