# Supplementary figures and images for: Genomic characterization and recombination analysis of hepatitis E virus in humans and swine across Asia: implications for food safety
Source: Front Microbiol. 2026 Mar 11;17:1744587. doi: 10.3389/fmicb.2026.1744587 (PMC13013505; doi:10.3389/fmicb.2026.1744587)

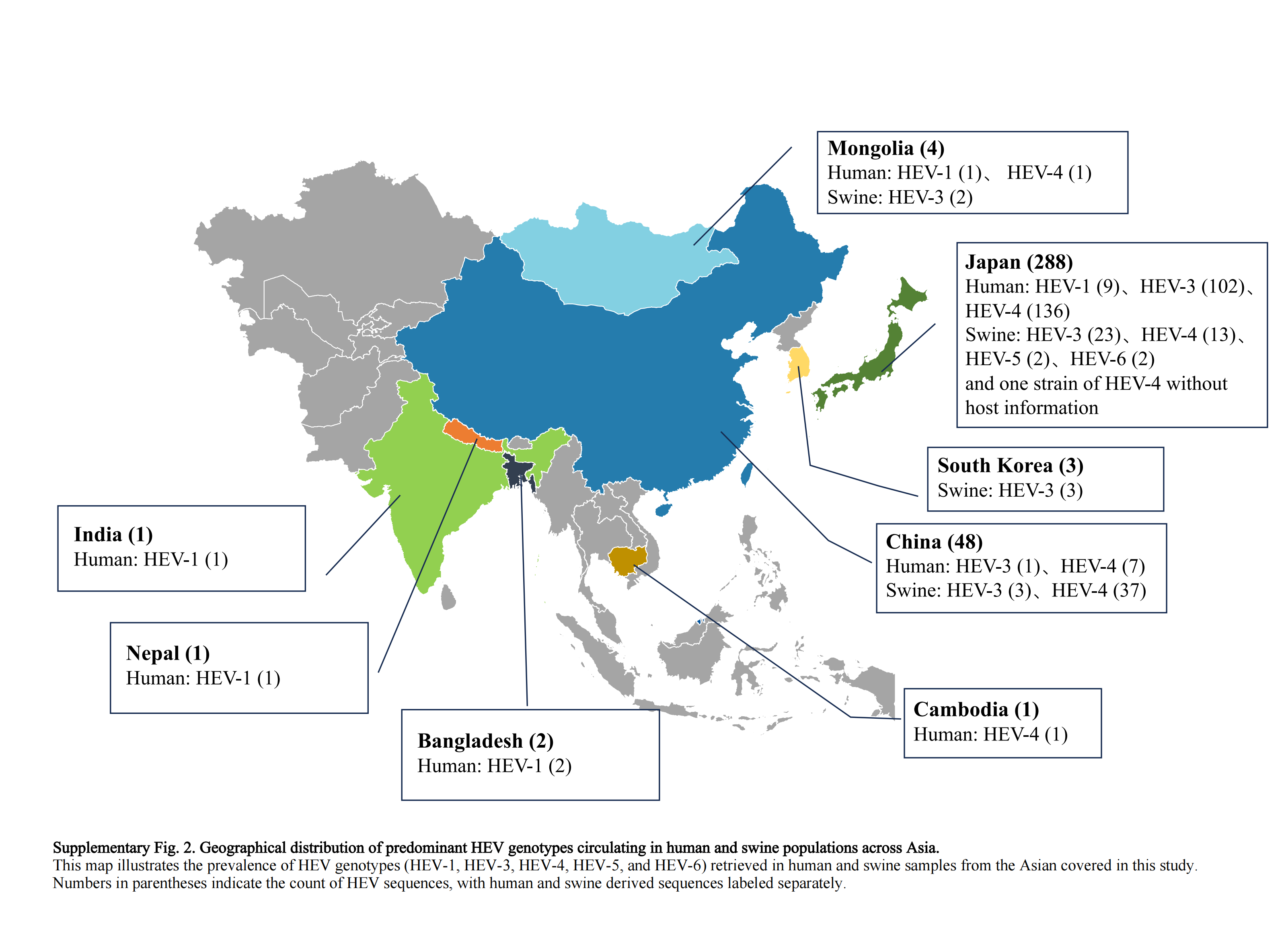

Supplement: Supplementary file 2 [file Image_2.tif]
